# Lactoferrin binding protein B – a bi-functional bacterial receptor protein

**DOI:** 10.1371/journal.ppat.1006244

**Published:** 2017-03-03

**Authors:** Nicholas K. H. Ostan, Rong-Hua Yu, Dixon Ng, Christine Chieh-Lin Lai, Anastassia K. Pogoutse, Vladimir Sarpe, Morgan Hepburn, Joey Sheff, Shaunak Raval, David C. Schriemer, Trevor F. Moraes, Anthony B. Schryvers

**Affiliations:** 1 Department of Microbiology & Infectious Diseases, University of Calgary, Calgary, Alberta, Canada; 2 Department of Biochemistry, University of Toronto, Toronto, Ontario, Canada; 3 Department of Biochemistry & Molecular Biology, University of Calgary, Calgary, Alberta, Canada; 4 Department of Chemistry, University of Calgary, Calgary, Alberta, Canada; University of Utah School of Medicine, UNITED STATES

## Abstract

Lactoferrin binding protein B (LbpB) is a bi-lobed outer membrane-bound lipoprotein that comprises part of the lactoferrin (Lf) receptor complex in *Neisseria meningitidis* and other Gram-negative pathogens. Recent studies have demonstrated that LbpB plays a role in protecting the bacteria from cationic antimicrobial peptides due to large regions rich in anionic residues in the C-terminal lobe. Relative to its homolog, transferrin-binding protein B (TbpB), there currently is little evidence for its role in iron acquisition and relatively little structural and biophysical information on its interaction with Lf. In this study, a combination of crosslinking and deuterium exchange coupled to mass spectrometry, information-driven computational docking, bio-layer interferometry, and site-directed mutagenesis was used to probe LbpB:hLf complexes. The formation of a 1:1 complex of iron-loaded Lf and LbpB involves an interaction between the Lf C-lobe and LbpB N-lobe, comparable to TbpB, consistent with a potential role in iron acquisition. The Lf N-lobe is also capable of binding to negatively charged regions of the LbpB C-lobe and possibly other sites such that a variety of higher order complexes are formed. Our results are consistent with LbpB serving dual roles focused primarily on iron acquisition when exposed to limited levels of iron-loaded Lf on the mucosal surface and effectively binding apo Lf when exposed to high levels at sites of inflammation.

## Author summary

Bacteria responsible for important infections in humans and food production animals survive and proliferate within their host by ‘hijacking’ iron from the host iron-binding proteins, transferrin and lactoferrin. The iron-hijacking process is mediated by a set of surface receptors that are specific for transferrin and lactoferrin from the host. In this study we focused on the receptors from important human pathogens responsible for meningitis and gonorrhea that are being targeted for development of vaccines, thus a detailed understanding of the structure and function of these proteins is needed to aid in vaccine design. Although there is detailed information available for the transferrin receptor proteins, currently information is lacking for the lactoferrin receptor proteins. This study focuses on a specific constituent of the lactoferrin receptor, lactoferrin binding protein B, that also serves to protect the bacteria against host defense mechanisms mediated by small peptides that kill microbes, including ones derived from host lactoferrin. We demonstrated that lactoferrin binding protein B has two different sites for binding lactoferrin, one associated with obtaining iron, the other related to protection against the antimicrobial peptides. This information enabled us to understand how this protein can effectively serve both roles and adapt to the local conditions.

## Introduction

*Neisseria meningitidis* is a diplococcal, Gram-negative bacteria that lives commensally in the nasopharyngeal tract of approximately 10–20% of humans [1]. *N*. *meningitidis* is an opportunistic pathogen that can cause serious invasive infections including meningitis and sepsis. This pathogen acquires iron–an essential cofactor required for redox reactions in biological processes–from the iron-loaded host glycoproteins, human transferrin (hTf) and human lactoferrin (hLf) using a set of specialized receptors with specific affinity for these host glycoproteins[2]. The transferrin and lactoferrin receptors from *N*. *meningitidis* are both comprised of an integral outer-membrane ‘A’ protein (TbpA, LbpA) and a bi-lobed, lipidated ‘B’ protein associated with the outer membrane (TbpB, LbpB). hTf is present in serum, within interstitial fluids and on mucosal surfaces, whereas hLf is localized to the mucosal surface, secretions, and sites of inflammation–possibly providing different niches for the functionality of these receptors [3].

**Fig 1:**
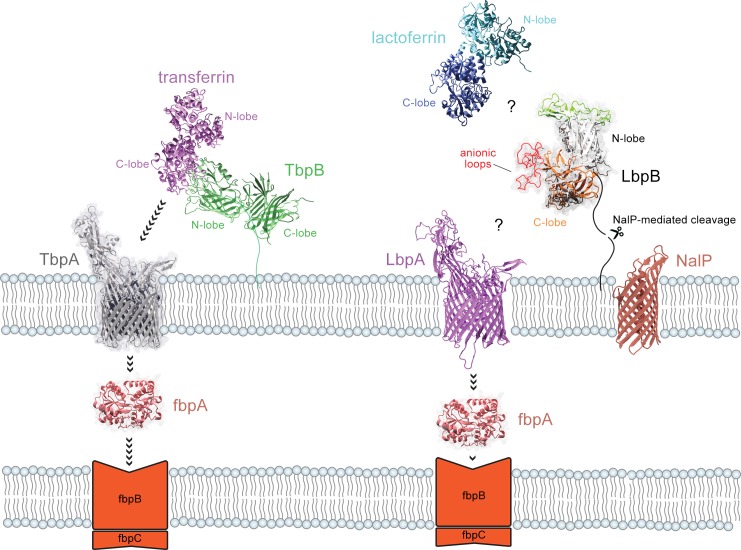
Model for iron acquisition from hTf and hLf. TbpB N-lobe captures iron-loaded hTf by binding to the C-lobe and transferring it to TbpA. TbpA extracts iron from hTf and transports it across the outer membrane where it is bound by FbpA and subsequently transported into the cytoplasm by the FbpBC inner membrane complex. It remains unclear whether hLf directly binds LbpA or is it first bound by LbpB, analogous to the TbpBA receptor or whether cleavage by NalP would compromise the role of LbpB in iron transport.

The molecular mechanism by which the transferrin receptor hijacks iron from hTf has been well studied from a structural and biophysical perspective. Crystal structures of TbpB, TbpB:hTf, and TbpA:hTf from *N*. *meningitidis* have all been determined [4, 5], providing an in-depth picture of the iron acquisition pathway (Fig 1). The N-lobe of TbpB captures the iron-loaded C-lobe of hTf and brings it to TbpA where iron is removed, transported across the outer membrane, captured by FbpA and transported into the cytoplasm through the FbpBC complex.

LbpA is required for acquiring iron from hLf [6] and binds to both domains of the C-lobe of hLf [7], suggesting that its removal of iron from hLf is analogous to the TbpA:Tf complex [5]. However, there is uncertainty in the interaction of LbpB with Lf and whether or not it plays a role in the iron acquisition process, particularly since it has been shown to be released from the meningococcal outer membrane by NalP [8] (Fig 1). Although the structures of the N-terminal lobe of LbpB from *N*. *meningitidis* and *Moraxella bovis* have been determined [9, 10], attempts to crystallize the intact LbpB protein from *N*. *meningitidis* have failed [10], likely due to large flexible clusters of anionic amino acids present in the C-terminal lobe. Computational docking with the structure of the *N*. *meningitidis* LbpB N-lobe predicted a binding interaction with the hLf N-lobe that contrasts the binding interface seen in the TbpB:hTf interaction. In contrast, Noinaj and Cornelisson et al. [11] proposed a model in which LbpB binds to the C-lobe of hLf. Neither of these models are based on experimental data and ignore the presence of LbpB’s defining characteristic–the presence of negatively charged loops in its C-lobe.

Recent work [12–14] has also implicated LbpB in defending the bacteria against neutrophil exudates and anti-microbial peptides (including human lactoferricin; a peptide proteolytically derived from human lactoferrin), indicating this protein may perform additional functions outside of iron-acquisition *in vivo–*or this may now be its primary or sole function. This function would not be compromised by the release of LbpB by NalP-mediated cleavage of the anchor peptide [8] but NalP-mediated release would impact iron acquisition if LbpB played a similar role as TbpB in the process. These observations, coupled with the conflicting models and incomplete molecular picture of LbpB call for an in-depth characterization of this protein if we are to understand *N*. *meningitidis* pathogenicity. In this study, we employ the use of several biophysical and biochemical strategies in order to characterize the interaction between LbpB and lactoferrin. We provide a more comprehensive picture of the binding mechanisms of LbpB to hLf and novel insights as to how the interactions may serve different roles under iron-limited and inflammatory conditions *N*. *meningitidis* would experience within the host.

## Results

### LbpB shares structural features with TbpB

In TbpB, the N-lobe and C-lobe are in a tight association with the two lobes in a perpendicular orientation [4, 15] that may serve an important functional role in the iron acquisition process. The published structures of the LbpB N-lobes [9, 10] confirm that the core structural features of the individual lobes, the antiparallel beta-strand handle and barrel domains, are conserved between TbpBs and LbpBs. However, the lack of success in obtaining structural information for intact LbpB and the uncertainty regarding its main physiological role raise questions as to whether LbpB shares the specific inter-lobe interactions with TbpB. To address this issue we initiated crosslinking coupled to mass spectrometry (XL-MS) experiments [16] to compare TbpB and LbpB. Although the identified intra-protein crosslinks will validate the overall structure of the individual lobes, our focus was on identifying inter-lobe crosslinks.

A crosslinking reagent was used that adds an 11.4Å spacer group between accessible primary amine groups situated on lysine residues which are located up to 30Å from one another. We treated full-length LbpB and TbpB from *N*. *meningitidis* strains MC58 and B16B6, respectively, with a homobifunctional N-hydroxysuccinimide ester crosslinker (disuccinimidyl suberate; DSS), and analyzed the trypsin-digested products via LC-MS/MS. LbpB crosslinks were mapped onto an *in silico* homology based model of LbpB using Swiss-Model that was modelled against *N*. *meningitidis* TbpB [4] (PDB entry 3V8U) as a template. The LbpB model should be quite reliable for the core beta-barrel and handle structures but will be least reliable in the loop regions that harbour the clusters rich in aspartic acid and glutamic acid residues. TbpB crosslinks were mapped onto the crystal structure of TbpB from *N*. *meningitidis* strain B16B6 [17] as a control. Distances between alpha carbons of crosslinked lysines were measured in PyMOL.

The structural models with mapped crosslinks are illustrated in S1 Fig and the details are provided in S1 Table and S2 Table. There is a single crosslink supporting the perpendicular association of the N-lobe and C-lobe in both the LbpB model (Panel A, red and blue residues) and TbpB model (Panel B, yellow residues), suggesting that LbpB shares the perpendicular association of the lobes with TbpB. Although the inter-lobe crosslink distance for LbpB is greater than the 30Å limit, it is influenced by the positioning of the large negatively charged C-lobe loops, the least reliable portion of the model. Thus it is not unreasonable to propose that the loops extend upwards near the N-lobe β-handle to fit within the crosslink limits. The only other crosslink which deviated from the 30Å limit was a crosslink between the LbpB-N barrel and anchor peptide. The variable positioning of the anchor peptide in structures of TbpB [15, 18] are consistent with flexibility that would result in the K26 to K287 crosslink.

### LbpB and TbpB show preference for the iron-loaded form of their cognate glycoprotein

**Fig 2:**
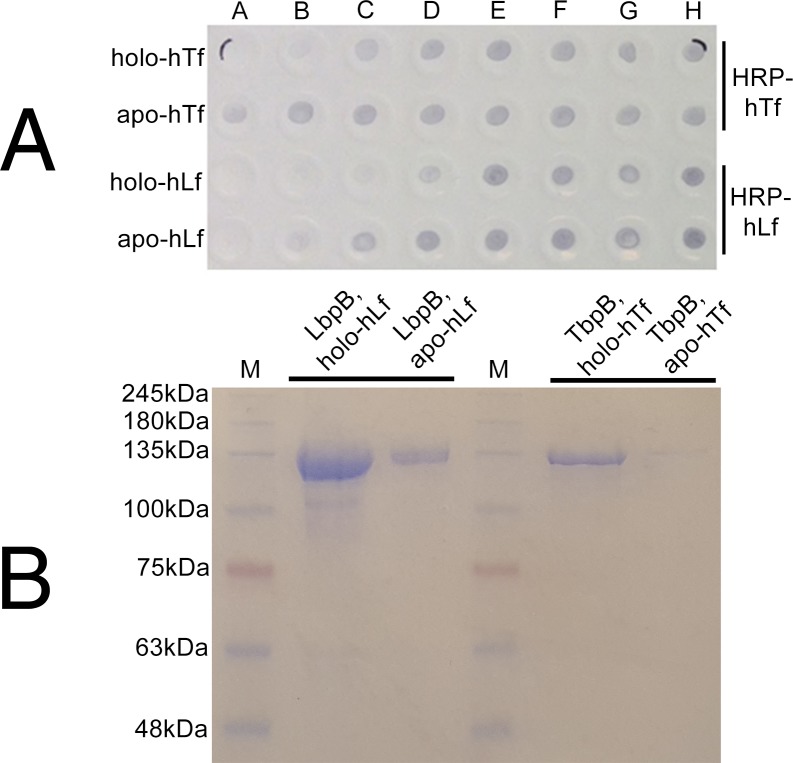
Specificity of LbpB and TbpB for iron-loaded glycoprotein. (A) Competitive solid-phase binding assay of TbpB with apo/holo hTf and LbpB with apo/holo hLf. Recombinant MBP-TbpB (top two rows) and MBP-LbpB (bottom two rows) were applied to nitrocellulose paper, the paper blocked and then incubated with apo- or holo- glycoprotein overnight in a ¼ serially diluted fashion (A, 20nM; B, 5nM; C, 1.25nM; D, 0.31nM; E, 0.07nM; F, 0.01nM; G, 4.88 × 10^-3^nM; H, 0nM). Iron-loaded HRP-conjugated glycoprotein (HRP-hTf or HRP-hLf) was then introduced into the binding mixture. Presence of a dot represents the displacement of any protein bound to TbpB or LbpB by the HRP-conjugate at the given concentration. (B) SDS-PAGE/affinity capture representing receptor protein (MBP-TbpB, 122kDa; or MBP-LbpB, 122kDa) captured by Sepharose resins conjugated to their cognate apo- or holo-glycoprotein (hTf-r, hLf-r).

TbpB has been shown to bind to both domains of the iron-loaded form of human Tf C-lobe, effectively trapping it in the closed conformation [4]. Thus, the preference of LbpB for holo or apo lactoferrin was examined, reasoning that it would implicate LbpB in binding to Lf in a similar fashion that TbpB binds to Tf. A competitive solid-phase binding assay was carried out in which increasing concentrations of the apo and holo forms of hTf or hLf were incubated with labeled hTf or hLf and immobilized TbpB or LbpB (Fig 2A). Both receptor proteins preferentially bound the holo form of their respective glycoprotein over the apo form in a competitive environment. However, apo-hLf was able to block binding of the labeled holo-hLf at high concentrations. Affinity capture experiments were performed in which LbpB and TbpB were incubated with hLf- and hTf-conjugated resins, respectively, in both iron-loaded (holo) and iron-free (apo) forms. Fig 2B indicates that both LbpB and TbpB preferentially bound the iron-loaded form of their cognate glycoprotein, though LbpB appears to have the capacity to bind apo-lactoferrin.

### LbpB harbors Lf binding capacity in both of its N- and C-terminal lobes

**Fig 3:**
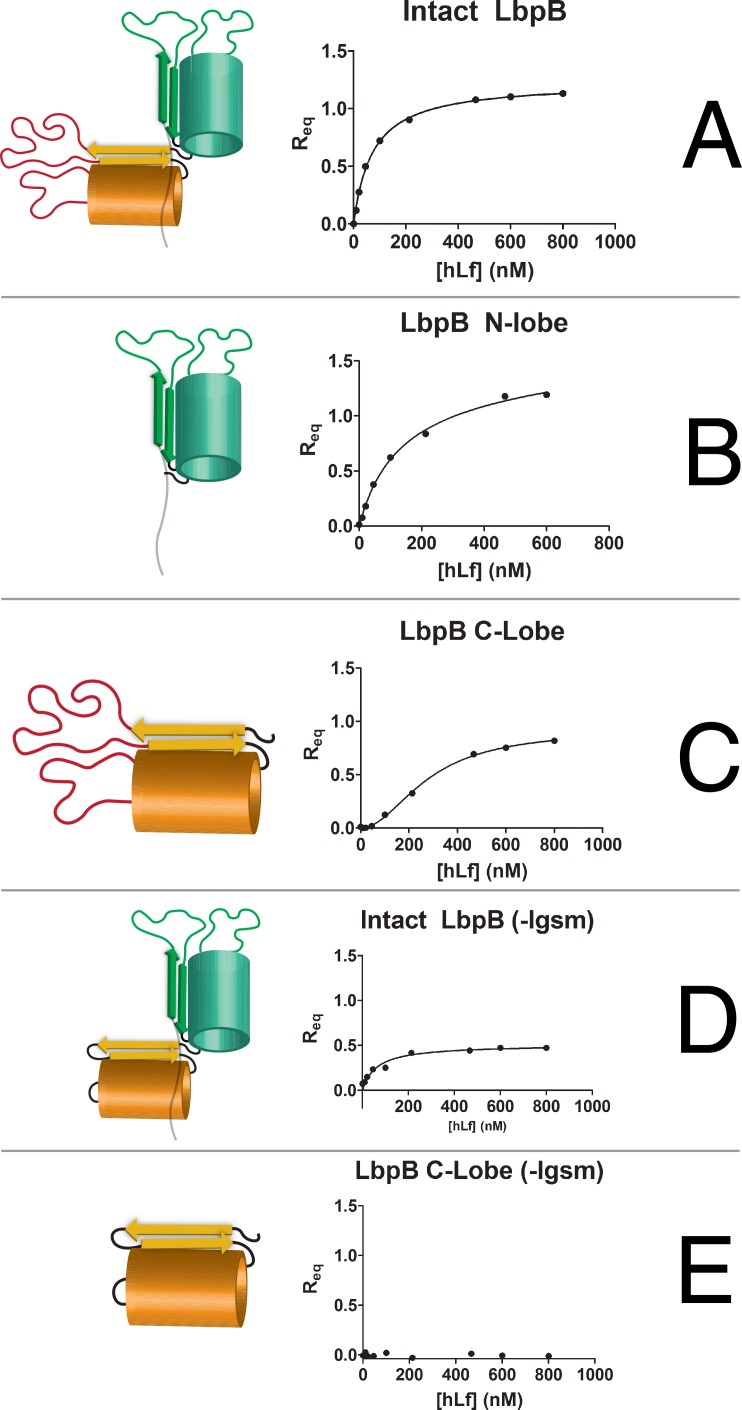
Receptor lobe binding contributions in TbpB and LbpB. Cartoon representations of each recombinant LbpB protein are displayed beside their respective BLI steady-state binding curve from binding hLf. (A) Intact LbpB, K_D app_ = 72.8 ± 3.24nM. (B) LbpB-N lobe, K_D app_ = 126 ± 48nM. (C) LbpB-C lobe K_D app_ = 279 ± 15nM. C-lobe Hill slope was calculated to be 1.98 ± 0.13 implying positive cooperativity. (D) Intact-lgsm, K_D app_ = 140 ±82.4nM (E). LbpB-C lobe-lgsm had no observed binding.

The demonstrated preference of LbpB for the iron-loaded form of Lf, implicates a similarity to TbpB that binds the iron-loaded C-lobe of Tf with its N-terminal lobe [15]. Similarly, the presence of regions rich in acidic amino acids in the C-terminal lobe capable of binding lactoferricin, suggests that the C-lobe would be capable of binding to the N-terminal lobe of Lf [2]. In order to address the binding properties of the individual LbpB lobes, we generated several recombinant domains of LbpB to tease apart binding affinities between each domain of LbpB with hLf using biolayer interferometry (BLI). Each recombinant protein harbored an N-terminal biotin acceptor peptide [19] that would mediate binding onto streptavidin-coated BLI sensors even from crude extracts [20]. These proteins included intact-LbpB, the N-terminal lobe, the C-terminal lobe, LbpB with the anionic loops removed (LbpB-lgsm; removal of amino acids 469–533 and 691–721), and the C-terminal lobe with the anionic loops removed (LbpB-C-lgsm). Cartoon representations of these proteins can be seen in Fig 3 alongside each of their respective steady-state binding curves with holo-hLf.

Binding activity was observed for intact LbpB and both of its constituent lobes, with the binding characteristics of the N-lobe more closely resembling that of intact LbpB (Fig 3). The LbpB-C-lobe yielded a sigmoidal steady state binding curve (Fig 3C). LbpB-C-lgsm showed no binding (Fig 3E), suggesting that the two anionic loops are responsible for the sigmoidal binding observed with the LbpB C-lobe. The observation that the C-lobe bound Lf with a relatively high K_D_ that was eliminated by removal of the anionic loops is consistent with the expectation that these loops mediated binding to the cationic region of the hLf N-lobe. Absence or differences in binding hLf cannot be attributed to problems with binding the LbpB derivatives as they were all observed to bind the streptavidin BLI sensors during the loading step (S2 Fig). To ensure the lack of binding seen from LbpB-C-lgsm to hLf was due to removal of the anionic loops and not simply misfolding, we performed intra-protein crosslinking on the recombinant protein and observed localized regions of crosslinks, that, when mapped onto a hypothetical model of LbpB-C-lgsm were all within the 30Å limit (S2A Fig, S3 Fig, S3 Table). Similarly, the observation that the LbpB N-lobe was capable of binding hLf with a K_D_ similar to intact LbpB with the anionic loops removed, is consistent with the proposal that the binding interaction is comparable to that observed with TbpB. Intact LbpB, which has binding contributions from both lobes, had a lower calculated K_D_ value than LbpB-lgsm, as would be expected.

### LbpB preferentially binds holo Lf with its predicted N-lobe binding site

The ability of LbpB to bind Lf at two different sites complicates the analysis of specific interactions at the individual sites. Our recent success at using XL-MS to probe the known Tf-TbpB interaction [21] prompted us to use XL-MS to capture individual LbpB:Lf complexes and analyze the composition and interactions involved. Similarly, our prior experience at probing the TbpB:Tf interaction with hydrogen/deuterium exchange coupled to mass spectrometry (HX-MS) [18, 22, 23] prompted us to use this complementary approach to characterize the LbpB:hLf interaction.

Since our BLI experiments were performed with LbpB fused to MBP, our initial attempts utilized MBP-LbpB in crosslinking reactions with hLf. Mixtures of MBP-LbpB and hLf were treated with DSS to covalently ligate lysines within the protein complexes and the individual complexes were isolated by SDS-PAGE for analysis (S4 Fig) by LC-MS/MS. Since we were not interested in characterizing the structure of MBP or hLf, and had already evaluated intra-protein crosslinks (S1 Table), only crosslinked peptides that included peptides derived from LbpB and hLf were selected for analysis. The peptides derived from 1:1 complexes were shown to contain linkages between the LbpB N-lobe and the hLf C-lobe (Table 1) indicating that this interaction predominated when holo-Lf was incubated with LbpB.

**Table 1:** Crosslinked lysines in the hLf:LbpB complex.

LbpB	hLf	Regions involved in crosslink
K118	K517	LbpB-N cap: hLf-C
K122	K517	LbpB-N cap: hLf-C
K140	K74	LbpB-N handle: hLf-N
K140	K334	LbpB-N cap: hLf-linker
K144	K334	LbpB-N cap: hLf-linker
K146	K620	LbpB-N cap: hLf-C
K146	K334	LbpB-N cap: hLf-linker
K151	K620	LbpB-N cap: hLf-C

For the HX-MS analysis we isolated LbpB after TEV cleavage of the MBP-LbpB preparation and also used this material for additional XL-MS experiments (S4 Fig). Preparations of hLf, LbpB and an hLf:LbpB complex were subjected to HX-MS analysis to evaluate the protein:protein interactions and conformational changes associated with complex formation. The data in S5 Fig illustrates that the main protection from deuterium was observed in peptides derived from the LbpB N-terminal lobe and the hLf C-terminal lobe (red boxes), consistent with an interaction predominantly involving these regions. It is important to note that there are substantial gaps in peptide coverage in both of these regions such that the full impact of binding might not be detected.

**Fig 4:**
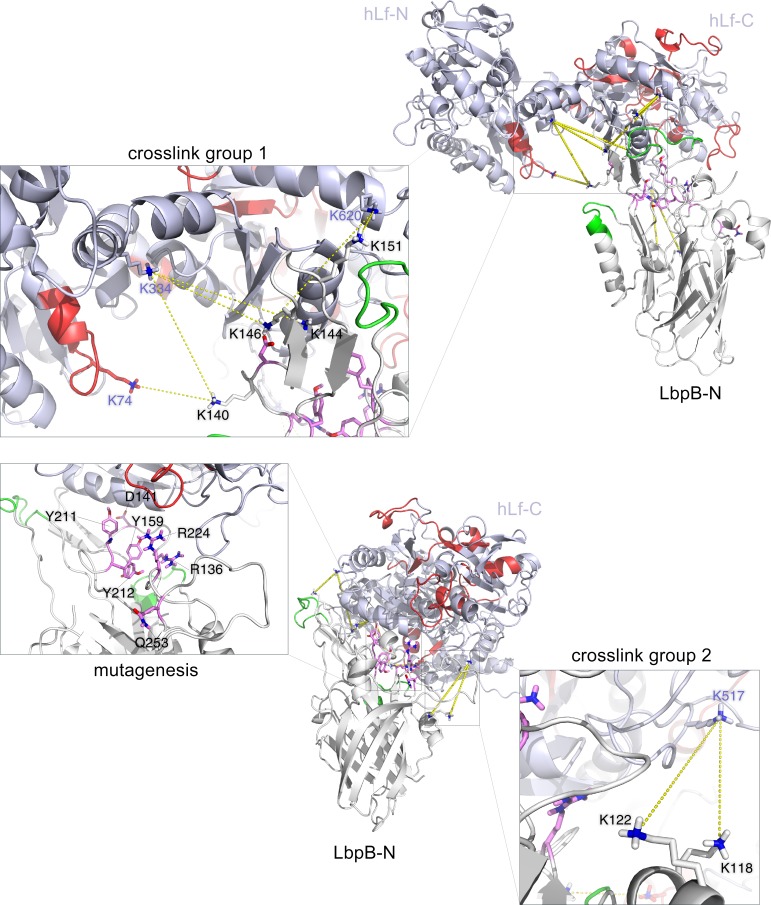
Data-directed docking model for the LbpB:hLf complex. The upper model shows the crystal structure of the LbpB-N lobe (white) docked to hLf (pale blue) on its C-terminal lobe. Green and red regions correspond to protected peptides in the LbpB:hLf complex (lower solvent accessibility) during HX-MS experiments for LbpB and hLf, respectively. Residues selected for mutagenesis which had an impact on complex formation are labelled in magenta. Crosslinks are indicated by yellow linker lines. An additional panel shows a zoomed-in view of one of two groups of crosslinks obtained from XL-MS studies (crosslink group 1). Lysines on hLf are labelled in a blue font, and LbpB lysines are labelled in a black font. The lower model is the same docked model as above, rotated 90 degrees on the Z-axis. A zoomed-in view of the second group of crosslinks (right side) and mutated residues (left side) are provided.

In order to gain a better sense of how the experimental data could be reconciled and the interaction visualized, we performed data-directed docking experiments with the LbpB-N lobe crystal structure from *N*. *meningitidis* (PDB entry 4U9C) and holo-hLf (PDB entry 2BJJ). To dock these two proteins, we used the HADDOCK 2.2 web interface and included the inter-protein constraints relevant to the LbpB-N:hLf-C interaction noted in Table 1. The crosslinked residues were converted to ambiguous restraints for docking with a distance constraint of 5-25Å between the alpha carbons. A model from the top scoring cluster was used to illustrate the XL-MS and HX-MS data (Fig 4) and select site-directed mutants to provide experimental support for the proposed interaction. We generated site-directed mutants of LbpB-N after manually and computationally assessing the residues likely to be actively involved in the LbpB-N:hLf-C interaction. Residues which were manually chosen were in agreement with residues predicted by the online hotspot predictor KFC2 [24, 25]. Six mutants were created (two double mutants, four single mutants) with mutations predicted to have different effects on binding affinity (Table 2). The mutated residues that resulted in loss in binding activity are indicated as magenta sticks in Fig 4.

**Table 2:** LbpB mutants.

Construct	WT Residue	Position	Mutant Residue	Expected Effect	Observed Effect
Mutant 1*	R	136	A	Binding ablation	No binding
	D	141	A		
Mutant 2	Y	159	G	Binding ablation	No binding
Mutant 3*	Y	211	G	Binding ablation	No binding
	Y	212	A		
Mutant 4	E	68	D	Equal or lower affinity	No effect
Mutant 5	R	224	E	Lower affinity	pH sensitive
Mutant 6	Q	253	N	Equal or lower affinity	Lower affinity

*Double mutant.

A selected set of lysine residues involved in crosslinking and their respective crosslinked partner are denoted with dashed yellow linker lines (Fig 4). Notably several lysine residues at the edge of the LbpB:hLf interaction form crosslinks with several different regions of hLf (Table 1) which provided useful information for the data-driven computational docking. The regions of LbpB with reduced exchange of deuterium in complex with hLf are colored in green and the regions of hLf with reduced exchange of deuterium in complex with LbpB are colored in red. Notably there are substantial gaps in the coverage of peptides in the HX-MS analyses (S5 Fig) so the limited presence of green or red colored loops in the LbpB:hLf interface is not surprising. The substantial amount of red labeling in the hLf regions at the base of the iron binding cleft away from the binding interface is reminiscent of what was observed in HX-MS analyses of the TbpB-hTf interaction [18, 23] and likely reflects conformational constraints imposed by stabilizing the hLf C-lobe in the closed, iron-bound conformation.

The data-directed model for the LbpB:hLf complex is strikingly similar to the TbpB:hTf complex (S6A and S6B Fig) and is perhaps best illustrated in an overlay of the two structures (S6C Fig). S6D Fig shows the solid-phase binding assay results of each mutant beside the wild type LbpB control summarized in Table 2. Binding assays were performed in two different conditions (pH = 5.9, pH = 7.5) as we had noticed previously [26] that the affinity of LbpB for hLf varies with pH and notably the R224 mutant resulted in reduced binding at high pH.

### LbpB can bind Lf at different sites and form higher order complexes

In the experiments with LbpB and hLf, incubation with DSS followed by SDS-PAGE also resulted in the identification of a higher molecular weight species that was most consistent with a 2:1 hLf:LbpB complex (S4 Fig). The 260kDa band from experiments with the MBP-LbpB fusion protein was excised from the gel and trypsin-digested for analysis by LC-MS/MS. High scoring, redundant crosslinked peptides were obtained for internal LbpB peptides, internal Lf peptides, internal MBP peptides, peptides identified in Table 1 for the LbpB N-lobe:hLf C-lobe interaction and a peptide between LbpB residue K379 and hLf residue K39. This peptide represents a linkage between a residue at the base of the negatively charged loops in the LbpB C-lobe and the lactoferricin region of the hLf N-lobe, consistent with the binding activity observed with BLI (Fig 3).

In the various experiments we performed evaluating the formation of complexes between LbpB and hLf we commonly observed the formation of precipitates that were not observed in the control preparations with hLf or LbpB alone, particularly over longer incubation periods and with higher concentrations of the proteins. Although XL-MS and HX-MS were useful in characterizing the 1:1 complex between iron-loaded hLf and LbpB that could reflect the situation on the mucosal surface, their utility decreased substantially when dealing with higher order complexes that might occur at sites of inflammation where higher concentrations of apo hLf are present.

## Discussion

The ability to utilize host iron-binding glycoproteins as a source of iron for growth has been observed in Gram-negative bacteria that reside in the upper respiratory or genitourinary tracts of a variety of vertebrate hosts ranging from birds to humans [27, 28]. The relatively conserved composition and structural features of the bipartite Tf receptor amongst these diverse hosts argue for the presence of these receptors in bacteria that resided in ancestral hosts prior to the divergence of birds and mammals. The demonstration that positive selection in regions of Tf involved in binding to TbpA drove the evolution of transferrin responsible for the host specificity amongst these pathogens in primates [29], suggests that these same forces are responsible for the host specificity observed in pathogens from other vertebrate hosts [2, 30]. The ability to proliferate independently from other members of the upper respiratory or genitourinary microbial communities due to direct utilization of host Tf provides a selective advantage, to the extent that some bacteria have become dependent upon this mechanism for survival [31, 32]. The ability to directly use host Tf also enables these bacteria to proliferate in serum and interstitial tissue fluids inside the body, one reason that these bacteria are also important pathogens in their respective hosts.

The bipartite Lf receptors were likely derived from ancestral Tf receptors after establishment of the mammalian lineage and have also been shown to be important for growth and survival on the mucosal surface [3]. However, the *Neisseria* LbpB has been shown to protect against cationic peptides derived from the Lf N-terminal region [12, 13] and to be selectively released from the bacterial surface by NalP [8], raising questions about its role in the iron acquisition process. The fact that positive selection analysis of lactoferrin evolution in mammals primarily identified residues in the N-terminal lactoferricin and lactoferrampin regions [33], highlights the importance of the interaction of Lf with bacterial factors such as the Lf binding protein PspA [34] and LbpB. Computational docking was used to develop two recent models for the LbpB:Lf interaction that proposed that the LbpB N-lobe bound to the Lf C-lobe [11] or to the Lf N-lobe [10]. Since both models were developed without any supporting experimental data, it was important to implement experimental studies to probe the LbpB-Lf interaction.

**Fig 5:**
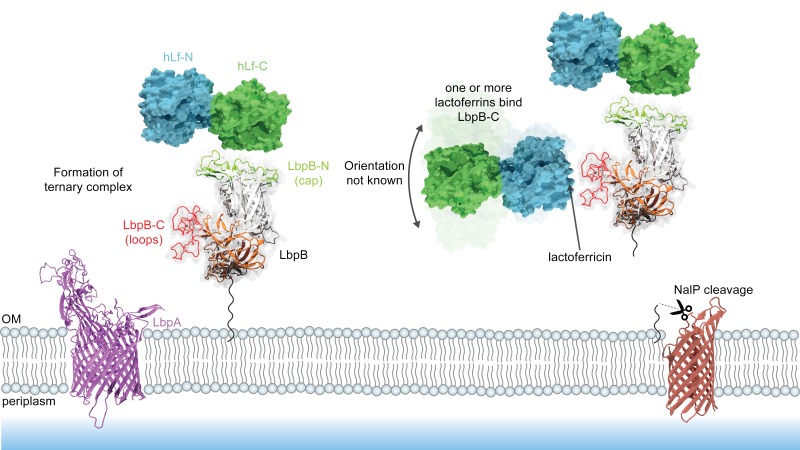
Proposed functions of LbpB. (LEFT) LbpB may be involved in the iron-acquisition pathway. At low concentrations of holo-hLf, LbpB may use its LbpB-N binding mode to preferentially bind iron-loaded lactoferrin and shuttle it to LbpA, forming a ternary complex and hijacking the iron. (RIGHT) Cleavage of LbpB from the membrane may be dependent on the presence of high levels of hLf in the extracellular milieu or simply a constitutive property of *N*. *meningitidis* cells in the NalP phase-variable ON-state. The release of LbpB from the membrane is done in an effort to sequester lactoferricin, antibodies, and possibly form large lattices of hLf as to prevent proteolytical processing into its derivative cationic antimicrobial peptides.

In this study the combined results from the biolayer interferometry binding studies (Fig 3), crosslinking coupled to mass spectrometry (XL-MS) and hydrogen/deuterium exchange coupled to mass spectrometry (HX-MS) (Fig 5) clearly indicate that the LbpB N-lobe binds to the hLf C-lobe analogous to what has been demonstrated for the Tf-TbpB interaction [4], thus supporting its potential role in iron acquisition. Furthermore, our crosslinking studies support the orthogonal orientation of the N-lobe and C-lobe (S1 Fig, S1 Table, S2 Table) and preferential association of the anchor peptide with the C-lobe (S1 Fig, S1 Table), features that have been proposed to be important for the transfer of iron-loaded Tf to TbpA [35]. Although hLf is secreted from mucosal glandular cells in the apo form, due to its ability to sequester iron, there may be a substantial proportion of holo (iron-loaded) hLf at the relatively low levels present under normal conditions on the mucosal surface [3]. Similarly, during invasive infection within the bloodstream hLf may predominantly be in the iron-loaded form. The ability to discriminate between the holo and apo forms of hLf (Fig 2) suggests that LbpB could serve an analogous function as TbpB by preferentially capturing holo-hLf under these conditions. Since the prevalence of the NalP gene is estimated to only be 88% in carriage and invasive isolates and the gene is subjected to phase variation by the presence of poly C tracts [36], there would be opportunities to select for isolates with efficient acquisition from hLf under these conditions.

LbpB has been shown to be important for protecting *N*. *meningitidis* against the killing activity of cationic antimicrobial peptides derived from hLf [12], and that this protection is mediated by the regions enriched in negatively charged residues in the C-lobe [13]. The results of our binding studies (Fig 3) and crosslinking studies confirm that the N-lobe of hLf is bound by the negatively charged regions in the C-lobe of LbpB. We only observed a single redundant crosslink between the LbpB-C lobe and hLf-N lobe, though this low level of coverage was to be expected considering the chemical nature of the crosslinker (reacts with primary amines) and the anionic nature of the LbpB-C lobe loops. Further studies may utilize additional crosslinker chemistries (i.e. carboxyl-to-amine crosslinkers) to address this issue. Since activated neutrophils secrete large concentrations of apo-Lf, this mode of binding may be particularly important at sites of inflammation, potentially inhibiting the release of cationic antimicrobial peptides by proteolytic cleavage of apo Lf or directly complexing the released cationic peptides. The release of LbpB by NalP would not necessarily compromise this process, and could potentially facilitate the formation of large Lf-LbpB complexes that might delay the release of cationic peptides. Since NalP has been shown to mediate the release of LbpB and the cleavage of human complement C3 [37], its expression upon crossing the mucosal barrier or at sites of inflammation would be advantageous.

This study has demonstrated the duality and complexity of the LbpB:Lf interaction that reflects the dual roles that LbpB plays in iron acquisition and protection from cationic peptides. A schematic for the proposed pathways can be seen in Fig 5. Considering the potential impact that LbpB release from the cell surface by selective NalP cleavage has on these two functions, there could be strong selective forces on NalP-on or NalP-off phase variants in different environments within the host. It would also be interesting to determine whether there may be other factors affecting the expression of NalP at sites of inflammation.

## Materials and methods

### Expression and purification of recombinant proteins

Regions of the *tbpB* and *lbpB* genes from *Neisseria meningitidis* strain MC58 or B16B6 were PCR amplified and cloned into a custom expression vector encoding a N-terminal polyhistidine tagged maltose binding protein followed by a TEV cleavage site [15]. The amplified gene regions not only excluded the signal peptide, but different segments of the N-terminus anchor peptide region that starts with the cysteine that is normally lipidated for anchoring the protein in the outer membrane. *N*. *meningitidis* (*N*.*m*.) strain M982 TbpB protein used in affinity-capture or BLI experiments excluded amino acids 1–40. *N*.*m*. MC58 LbpB protein excluded amino acids 1–15, and LbpB N-lobe protein excluded amino acids 1–35.

The recombinant plasmids were used to transform *Escherichia coli* strain ER2556 and after 1 hour incubation in LB broth containing 100 μM ampicillin, 1 mL was directly inoculated into a 20 mL starter culture of LB with ampicillin. After growth at 37°C for 18 hours the cells were re-inoculated into a 1 L culture of ZY auto-induction media containing ampicillin and allowed to grow for 24 hours. Cells were pelleted by centrifugation at 11,200 × RCF, the broth supernatant was decanted and cell pellets were re-suspended in 50 mM sodium phosphate, 300 mM NaCl, 5 mM imidazole, pH 7.4 buffer (resuspension buffer) and lysed using an Avestin Emulsiflex-C3 homogenizer. Lysates were centrifuged at 48,200 × RCF for 1 hour and the supernatant was filtered through a 0.45μm syringe filter. The filtered sample was loaded onto a 5 mL HisTrap FF column (GE Healthcare) using an Amersham peristaltic pump at a flow rate of 2 mL/min. The column was then loaded onto an AKTA purifier, washed in resuspension buffer followed by wash buffer (50 mM sodium phosphate, 300 mM NaCl, 30 mM imidazole, pH 7.4) until UV signal baselined. The target protein was eluted with elution buffer (50 mM sodium phosphate, 300 mM NaCl, 250 mM imidazole, pH 7.4) and selected fractions were pooled and dialyzed into interaction buffer (50 mM Tris-HCl, 50mM NaCl, pH 7.4) and stored at 4°C.

LbpB fused with MBP was released by TEV protease cleavage (Qiagen, in-house purified) overnight. MBP and LbpB were separated by ion exchange on a Q HP column (GE Healthcare) and the samples were eluted on a salt gradient (50 mM sodium phosphate, 2 M NaCl, pH 7.4). The LbpB containing fractions were dialyzed into interaction buffer and stored at 4°C.

### Solid-phase binding assays

3 μl of receptor protein preparations (recombinant MBP fusions) at concentrations of 1 mg/mL were applied to a nitrocellulose membrane (Pall, Hessen, Germany) and allowed to dry. The membrane was then blocked with a 1% skim milk (W/V) in SPB buffer (20 mM sodium phosphate, 150 mM NaCl, pH 5.9 or 20 mM Tris-HCl, 150 mM NaCl, pH 7.5) for 1 hour. A 1:1000 dilution of HRP-conjugated ligand at an approximate concentration of 1 mg/mL was added to the blocking solution, and incubated overnight, shaking at 4°C. The blocking solution was removed, and the membrane was washed three times for 5 min with SPB Buffer. A development stock solution was created from 300 mg of HRP Color Development Reagent (BioRad) in 100mL methanol, then stored at -20°C. A diluted form of the development reagent stock was used on the membrane (20 mL SPB buffer, 4 mL color development reagent stock, 200 μL H_2_O_2_).

### Affinity capture experiments

hTf and hLf-conjugated resins were generated using cyanogen bromide activated Sepharose 4B resin (GE Healthcare). Iron-free (apo) resins were generated by washing iron-loaded resin in a low pH buffer (1% sodium citrate, 100 mM EDTA, pH 3.0). 100 μL of 50% slurry was washed three times with binding buffer (10 mM sodium phosphate, 100 mM NaCl, pH 6.0) by centrifuging the resin at 6,000 × RCF for 1 min, decanting the supernatant, and resuspending the resin in binding buffer. Approximately 100 μg of free LbpB or TbpB protein was incubated with the resin for 2 hours at RT. Samples were centrifuged at 6,000 × RCF for 1 min, and supernatant was decanted. Resins were then washed three times in binding buffer with salt adjusted to 120 mM (hLf resin) or 1 M (hTf resin) for 20 min. 85 μL of 2 x SDS-loading buffer (100 mM Tris-HCl, 4% SDS, 0.2% bromophenol blue, 20% glycerol) was added to the resin, and boiled for 5 min to release bound protein.

### Biolayer interferometry

BLI experiments were carried as outlined in [20] on a ForteBio Octet RED 96 machine (Pall Biosciences). *E*. *coli* ER2566 cells were transformed with plasmids encoding the various recombinant protein constructs including a BirA biotin acceptor peptide (BAP) [19], and plated on LB-Agar plates. A set of 5 colonies were selected from each plate and inoculated into 5mL of auto-induction media. Cells were grown for 18 hours at 37°C. Bacterial pellets were resuspended in lysis buffer (1 X PBS, 1% Triton X-100). Lysozyme, DNase and PMSF were added to aid in lysis, decrease viscosity and prevent proteolysis. Resuspensions were incubated at room temperature for 15 min, and spun down in a table-top centrifuge at 16,100 × RCF for 25 min. The concentrations of hLf for the dilution series were chosen as to flank the K_D_ value for the TbpB:hTf interactions (~50 nM) by approximately tenfold in each direction. Concentration values were calculated on a logarithmic scale incrementing by 0.33 to obtain the values 10 nM, 21 nM, 46 nM, 100 nM, 213 nM, 467 nM, 600 nM and 800 nM. After an initial baseline in 1 X kinetics buffer (1 X PBS pH 7.4, 0.002% Tween-20, 0.1 mg/mL BSA) and sensor loading, assay steps followed a repeat pattern of: baseline, association, dissociation, and regeneration. Regeneration was carried out in a 100 mM sodium citrate, 50 mM EDTA, pH 4.5 buffer. Association and dissociation steps were carried out in kinetics buffer. Steady state values were obtained by averaging the response values obtained in the last 5 seconds of the association step were plotted against concentration to generate saturation binding curves, and the data was fitted using Prism (Graphpad). The “One site–Total binding” saturation curve was used for Intact, N-lobe and Intact-lgsm, whereas the “One site–specific binding with Hill slope” was used for C-lobe binding.

### Site-directed mutagenesis

Mutants were generated using splicing by overlap extension (SOE) PCR [38]. Primers harboring the desired mutations were incubated with vector-specific primers on the 5’ and 3’ end of the insertion site, and vector containing wild-type LbpB was used as template. Amplicons were then spliced together to create the recombinant mutant.

### XL-MS

*N*.*m*. MC58 LbpB and hLf (Agennix) were incubated together in interaction buffer at equimolar concentrations in a total volume of 50 μL, shaking gently for 4 hours at RT. A stock solution of disuccinimidyl suberate (DSS, Thermo Fisher Scientific) at 25 mM in DMSO was prepared and added to the complexed LbpB:hLf mixture to a final concentration of 1 mM. This mixture was gently mixed and incubated a further 30 min at room temperature. The DSS was then quenched using a 1 M NH_4_HCO_3_ solution added to a final concentration of 50 mM. Samples were loaded on a handcast 6% SDS-polyacrylamide gel until the 190kDa and 260kDa bands were completely separated from all other protein bands. The gel was stained with Coomassie blue, and the band of interest was excised with a clean scalpel blade for processing by conventional tryptic in-gel digestion methods [39], followed by analysis using an Orbitrap Velos mass spectrometer, equipped with an EasyLC1000 nanochromatography system (Thermo Scientific). Briefly, digests were reconstituted in 0.1% formic acid and loaded on an 8 cm x 75 μm self-packed picotip column (Aeris Peptide XB-C18, 3.6 μm particle size, Phenomenex). Separation was achieved using a 30 min 5–60% gradient of mobile phase B (97% acetonitrile with 0.1% formic acid) at 300 nl/minute. Mobile phase A consisted of 3% acetonitrile and 0.1% formic acid. The mass spectrometer was operated in positive ion mode, with a high/high configuration, where MS resolution set at 60,000 (400–2000 m/z) and MS^2^ resolution at 7500. Up to ten of the most abundant ions were selected for fragmentation using higher energy collisional dissociation (HCD), rejecting charge states 1 and 2, and using a normalized collision energy of 40%. Data analysis was performed using the crosslinking plugin within Mass Spec Studio 2.0, using default settings [21] with high-scoring (score of 12 or higher), and redundancy (observed in duplicate or greater) used as criteria for selection of crosslinks. Each crosslinked peptide pair was manually inspected for data quality and correct assignment of linked residues, using the manual validation user interface in the Studio. The final crosslinked peptides as well as each unique pair of crosslinked residues were exported in.CSV format for use in molecular docking.

### Molecular docking experiments

HADDOCK (High Ambiguity Driven protein-protein DOCKing) [40, 41] was used to generate data-driven models of the Lf:LbpB interaction. The docking process consists of three successive steps (rigid body energy minimization, semi-flexible refinement and solvent refinement) where data can be invoked, in the form of distance restraints, at each step to inform the development of docked models. Data can be defined as ambiguous or unambiguous, reflecting a degree of uncertainty in the correspondence of residues across an interface. Although this correspondence across the interface is determined by the crosslinking experiment, we chose to establish docking runs by treating crosslinked residues as ambiguous restraints, overlaid upon conventional center-of-mass restraints typically used in *ab initio* docking. This is approach is justified, as crosslinking data derived from DSS-based experiments typically generate a modest number of restraints, with poorly defined distances between linked residues. For docking we allowed crosslinks to vary between 5-25Å [42], and generated docking runs on the Haddock 2.2 webserver, using default settings (10,000 samplings in *it0*, 200 in *it1* and 200 during refinement in explicit solvent). Results were clustered according to fraction of common contacts (FCC), and the top-ranked cluster according to HADDOCK score was selected. Within the best cluster, the best-scoring model was chosen to represent the hLf:LbpB interaction.

### HX-MS

Stock solutions of hLf (20 μM) and LbpB (10 μM) were diluted to 5 μM in 10 mM Tris-HCl (pH 7.4) buffer prior to the HX-MS experiment. Similarly, the hLf:LbpB complex solution was prepared from the stock solutions in a 10 mM Tris-HCl (pH 7.4) buffer at a 1:1 ratio of hLf and LbpB with final concentration of 5 μM for each component protein. The complex solution was incubated for 1 hour prior to performing HX-MS analysis. HX was initiated by adding 2 μL diluted protein solutions to the labelling solution (90% D_2_O, in 10 mM Tris-HCl) at 4°C to a final D_2_O level of 45%. Labelling was performed for 1, 10, and 100 min for individual protein and the complex solutions. At the end of the labelling period, the samples were incubated in 100 mM TCEP under quenching conditions (100 mM glycine, pH 2.5) for 1 minute, followed by digestion at 10°C with 6 μL of recombinant NepII (0.1μg/mL, in 100mM Gly-HCl, pH 2.5) for 2 min [43]. The digested samples were loaded onto a self-packed preconcentration cartridge (25 mm x 250 μm i.d. capillary, 200 Å, 5 μm Magic C18 beads, Michrom BioResources) for 3 min (10 μL/min) at 4°C and separated on a self-packed analytical column (70 mm x 150 μm i.d. capillary, 200 Å, 5 μm Magic C18 beads using a 10–40% gradient over 10 min). HX data for all labelling time points were collected in triplicates with a TripleTOF 5600 (SCIEX) coupled to an Eksigent nanoLC-ultra-2D pump.

Mascot v2.4 was used to identify peptides for analysis. Briefly, a search was performed against a database containing all proteins present in this study with a mass tolerance for precursor ions of 20 ppm, 0.05 Da for fragment ions, and a probability cutoff of p = 0.05. A peptide list was next imported into our in-house software package MS Studio for deuteration analysis [44]. Peptide quality was assessed based on intensity, signal to noise ratio and spectral overlap, and only those with reliable isotopic profiles were selected for analysis. The percent relative fitted deuteration for each high-quality peptide was exported. Woods plots for each protein state were created using a statistical module in MS Studio as previously described [45].

## Supporting information

S1 Fig Crosslink mapping in TbpB and LbpB. (A) A two-sided view of LbpB and the intra-protein crosslinks between spherized lysine residues obtained using DSS. Groups of crosslinks within close proximity are grouped and labelled a unique colour (specified in Tables 1 and 2). Distances between alpha carbons atoms (in Å) are noted. (B) A two-sided view of TbpB and its intra-protein crosslinks.
(PDF)

S2 Fig Crosslinking of LbpB-C-lgsm and BLI sensor loading steps. (A) SDS-PAGE gel of the MBP-LbpB-C-lgsm in presence and absence of DSS (crosslinker). Arrows point to the band of interest in each case, and band-broadening upon addition of crosslinker can be seen, indicating successful crosslinking. (B) BLI sensor loading steps for each recombinant MBP-LbpB used in this study. Empty streptavidin sensors were placed in 1x kinetics buffer for 60 seconds before being placed in a crude preparation of biotinylated recombinant LbpBs. An increase in response (y-axis) with time (x-axis) indicates an increase in thickness of the biological layer on the sensor and thus protein binding.
(PDF)

S3 Fig Crosslink mapping in LbpB-C-lgsm. A model of LbpB-C-lgsm was generated using the sequence of LbpB-C with the anionic loops removed and modelled against TbpB, as no high-resolution structure is available for this protein. Intra-protein crosslinks were mapped onto the protein and localized regions of crosslinks were coloured with a unique colour.
(PDF)

S4 Fig SDS-PAGE of *LbpB*:*hLf complex populations*. TEV-cleaved LbpB and hLf were purified and loaded in lanes 1 and 2, respectively. An incubation of these two proteins in equal molar concentrations was then crosslinked with 2mM DSS. Crosslinked protein was concentrated and loaded in duplicate beside the marker (lane 3) in lanes 4 and 5. Appearance of a ~160kDa 1:1 complex, and ~245 kDa 2:1 complex are indicated.
(PDF)

S5 Fig HX-MS Woods plots for the interaction between hLf and LbpB. Difference in deuteration between (a) hLf-LbpB complex and free hLf and (b) hLf-LbpB complex and LbpB, plotted as a function of protein sequence. A reduction in deuteration, resulting from stabilization upon binding is shown in blue. Destabilization is shown in red. Peptides for which no significant change in deuteration was observed are shown in grey (p < 0.05). Dashed grey lines indicate the 2x SD deviation cut-off based on the error in all non-significant measured deuteration values.
(PDF)

S6 Fig Predicted complex structure of LbpB:hLf and mutagenesis studies. (A) Docked model of LbpB-N (PDB entry 4U9C, filled in with Swiss-Model) against diferric hLf (PDB entry 2BJJ) using XL-MS constraints. Binding interface is noted with a translucent gray rectangle. (B) Crystal structure of the TbpB-N:hTf-C interaction from *Neisseria meningitidis* M982 (PDB entry 3VE1). (C) Alignment of docked model from (A) with crystal structure from (B). (D) Solid phase binding assay of WT and mutant LbpBs binding hLf at pH 5.9 and 7.4.
(PDF)

S1 Table Intra-protein crosslinks for N.m. LbpB (MC58). * Inter-lobe crosslink.
**Low–medium confidence crosslink.
(PDF)

S2 Table Intra-protein crosslinks for N.m. TbpB (B16B6). * Inter-lobe crosslink.
(PDF)

S3 Table Intra-protein crosslinks for N.m. MBP-LbpB-C-lgsm (MC58). (PDF)
