# Supplementary material for: Lactoferrin binding protein B – a bi-functional bacterial receptor protein
Source: PLoS Pathog. 2017 Mar 3;13(3):e1006244. doi: 10.1371/journal.ppat.1006244 (PMC5352143; doi:10.1371/journal.ppat.1006244)
Supplement: S1 Fig — (A) A two-sided view of LbpB and the intra-protein crosslinks between spherized lysine residues obtained using DSS. Groups of crosslinks within close proximity are grouped and labelled a unique colour (specified in Tables 1 and 2). Distances between alpha carbons atoms (in Å) are noted. (B) A two-sided view of TbpB and its intra-protein crosslinks. (PDF) [file ppat.1006244.s001.pdf]

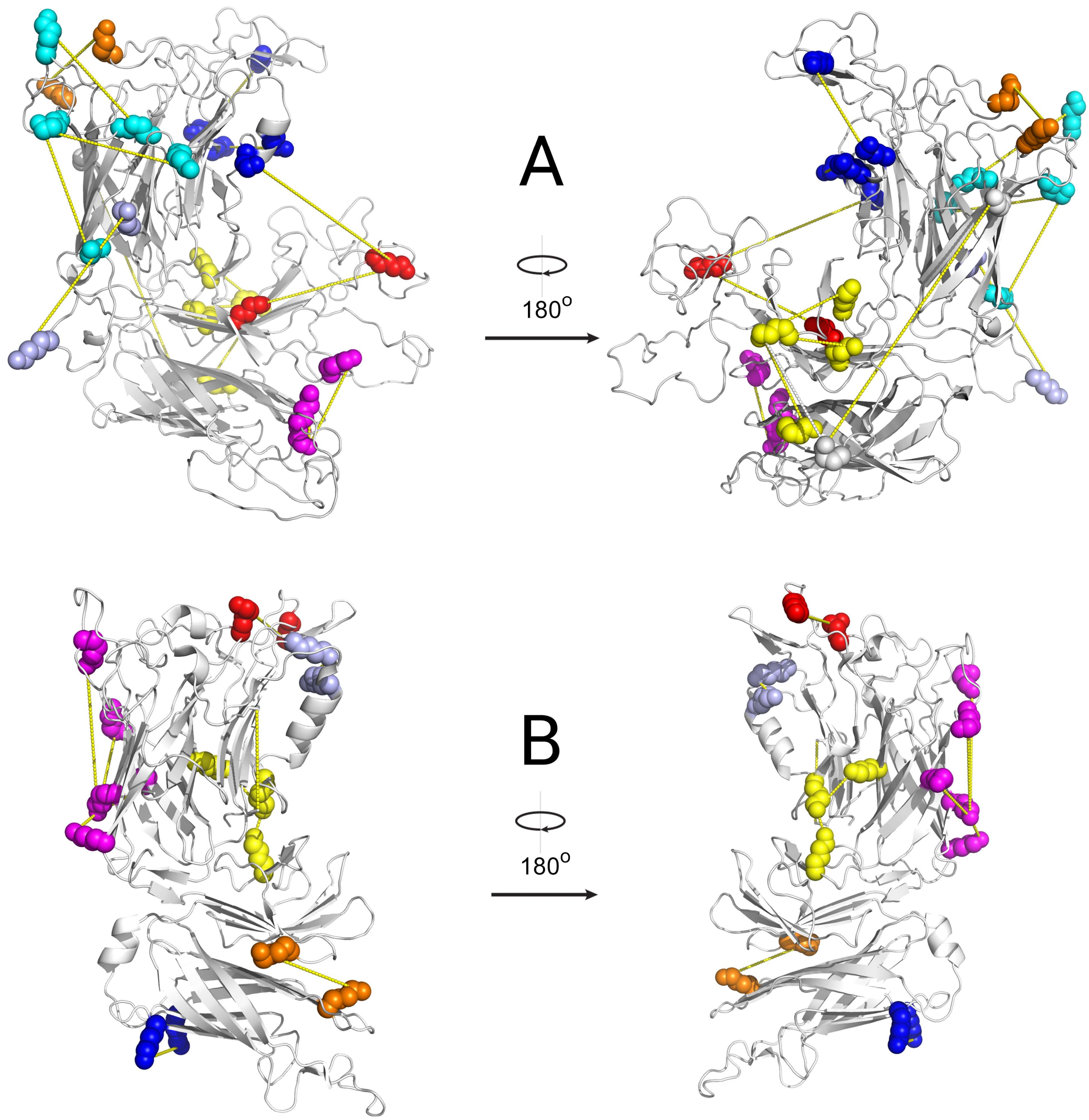

S1 Fig. *Crosslink mapping in TbpB and LbpB.* (A) A two-sided view of LbpB and the intra-protein crosslinks between spherized lysine residues obtained using DSS. Groups of crosslinks within close proximity are grouped and labelled a unique colour (specified in Tables 1 and 2). Distances between alpha carbons atoms (in Å) are noted. (B) A two-sided view of TbpB and its intra-protein crosslinks.
