# Supplementary material for: Lactoferrin binding protein B – a bi-functional bacterial receptor protein
Source: PLoS Pathog. 2017 Mar 3;13(3):e1006244. doi: 10.1371/journal.ppat.1006244 (PMC5352143; doi:10.1371/journal.ppat.1006244)
Supplement: S2 Fig — (A) SDS-PAGE gel of the MBP-LbpB-C-lgsm in presence and absence of DSS (crosslinker). Arrows point to the band of interest in each case, and band-broadening upon addition of crosslinker can be seen, indicating successful crosslinking. (B) BLI sensor loading steps for each recombinant MBP-LbpB used in this study. Empty streptavidin sensors were placed in 1x kinetics buffer for 60 seconds before being placed in a crude preparation of biotinylated recombinant LbpBs. An increase in response (y-axis) with time (x-axis) indicates an increase in thickness of the biological layer on the sensor and thus protein binding. (PDF) [file ppat.1006244.s002.pdf]

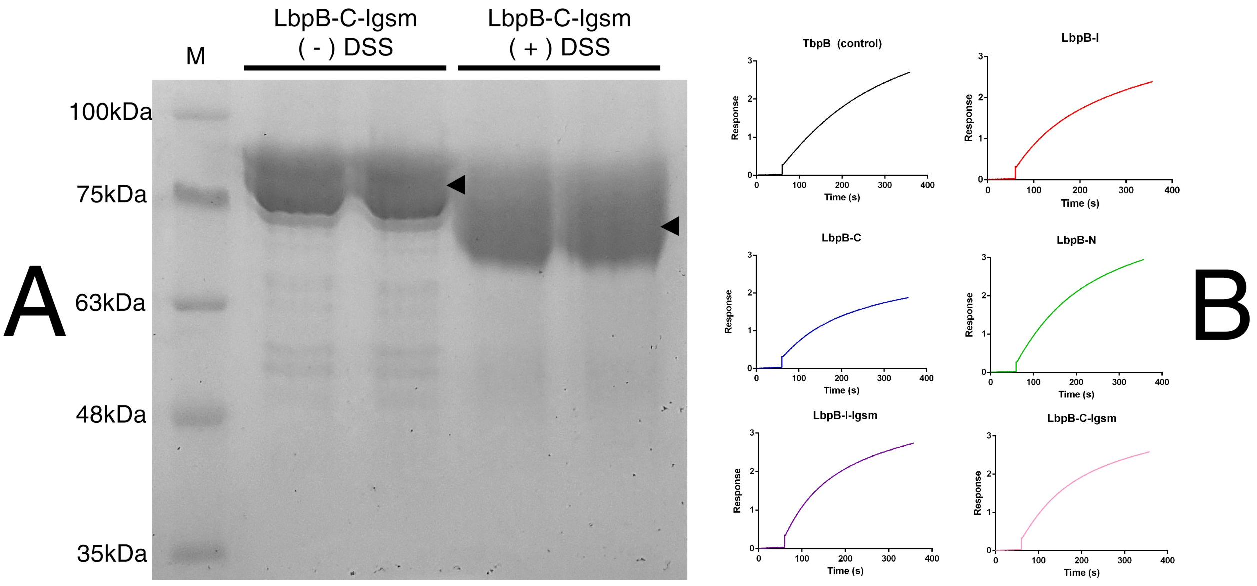


**S2 Fig.** *Crosslinking of LbpB-C-lgsm and BLI sensor loading steps.* (A) SDS-PAGE gel of the MBP-LbpB-C-lgsm in presence and absence of DSS (crosslinker). Arrows point to the band of interest in each case, and band-broadening upon addition of crosslinker can be seen, indicating successful crosslinking. (B) BLI sensor loading steps for each recombinant MBP-LbpB used in this study. Empty streptavidin sensors were placed in 1x kinetics buffer for 60 seconds before being placed in a crude preparation of biotinylated recombinant LbpBs. An increase in response (y-axis) with time (x-axis) indicates an increase in thickness of the biological layer on the sensor and thus protein binding.
