# Supplementary material for: Lactoferrin binding protein B – a bi-functional bacterial receptor protein
Source: PLoS Pathog. 2017 Mar 3;13(3):e1006244. doi: 10.1371/journal.ppat.1006244 (PMC5352143; doi:10.1371/journal.ppat.1006244)
Supplement: S3 Fig — A model of LbpB-C-lgsm was generated using the sequence of LbpB-C with the anionic loops removed and modelled against TbpB, as no high-resolution structure is available for this protein. Intra-protein crosslinks were mapped onto the protein and localized regions of crosslinks were coloured with a unique colour. (PDF) [file ppat.1006244.s003.pdf]

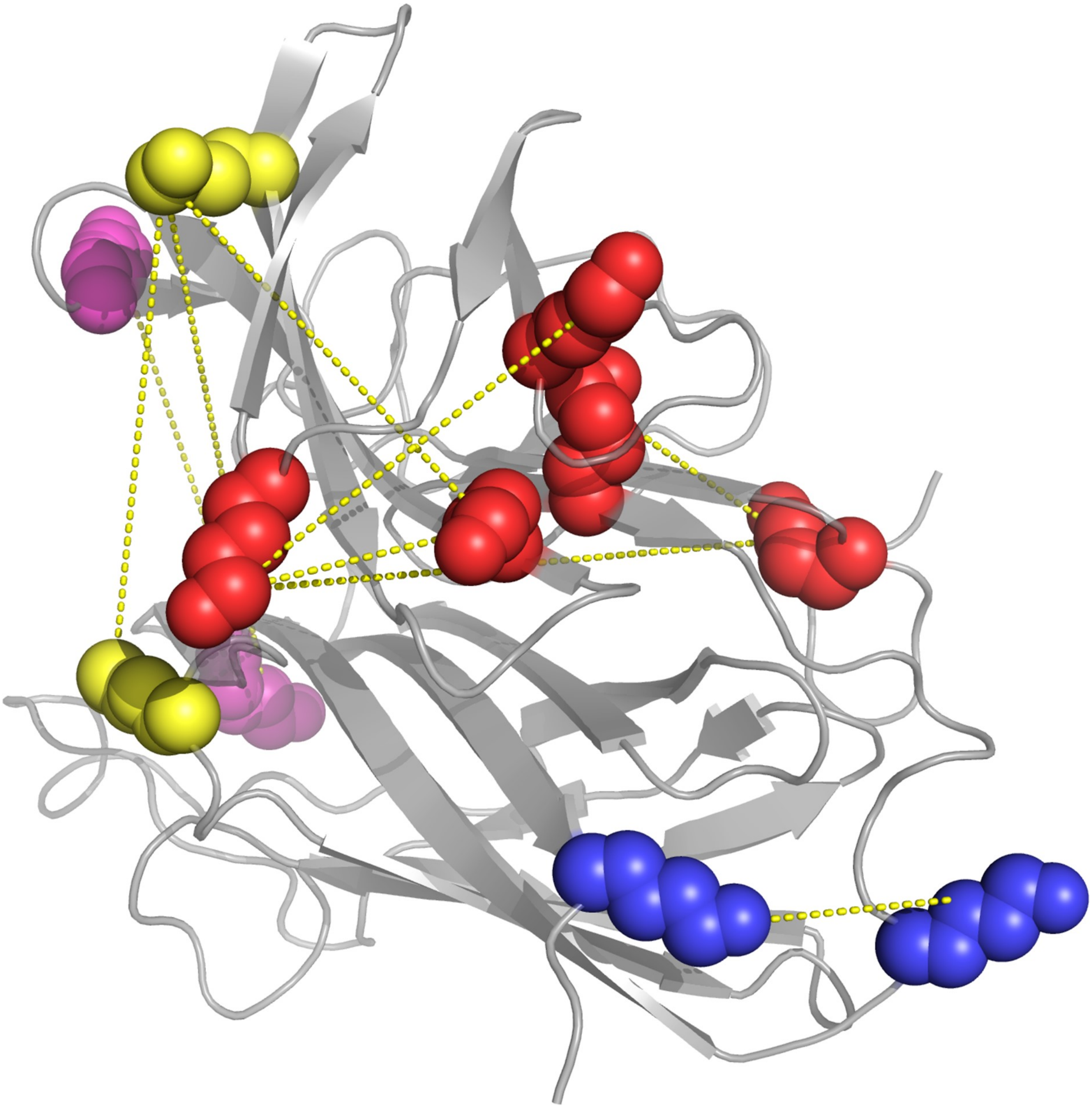

S3 Fig. *Crosslink mapping in LbpB-C-lgsm.* A model of LbpB-C-lgsm was generated using the sequence of LbpB-C with the anionic loops removed and modelled against TbpB, as no high-resolution structure is available for this protein. Intra-protein crosslinks were mapped onto the protein and localized regions of crosslinks were coloured with a unique colour.
