# Supplementary material for: Lactoferrin binding protein B – a bi-functional bacterial receptor protein
Source: PLoS Pathog. 2017 Mar 3;13(3):e1006244. doi: 10.1371/journal.ppat.1006244 (PMC5352143; doi:10.1371/journal.ppat.1006244)
Supplement: S4 Fig — TEV-cleaved LbpB and hLf were purified and loaded in lanes 1 and 2, respectively. An incubation of these two proteins in equal molar concentrations was then crosslinked with 2mM DSS. Crosslinked protein was concentrated and loaded in duplicate beside the marker (lane 3) in lanes 4 and 5. Appearance of a ~160kDa 1:1 complex, and ~245 kDa 2:1 complex are indicated. (PDF) [file ppat.1006244.s004.pdf]

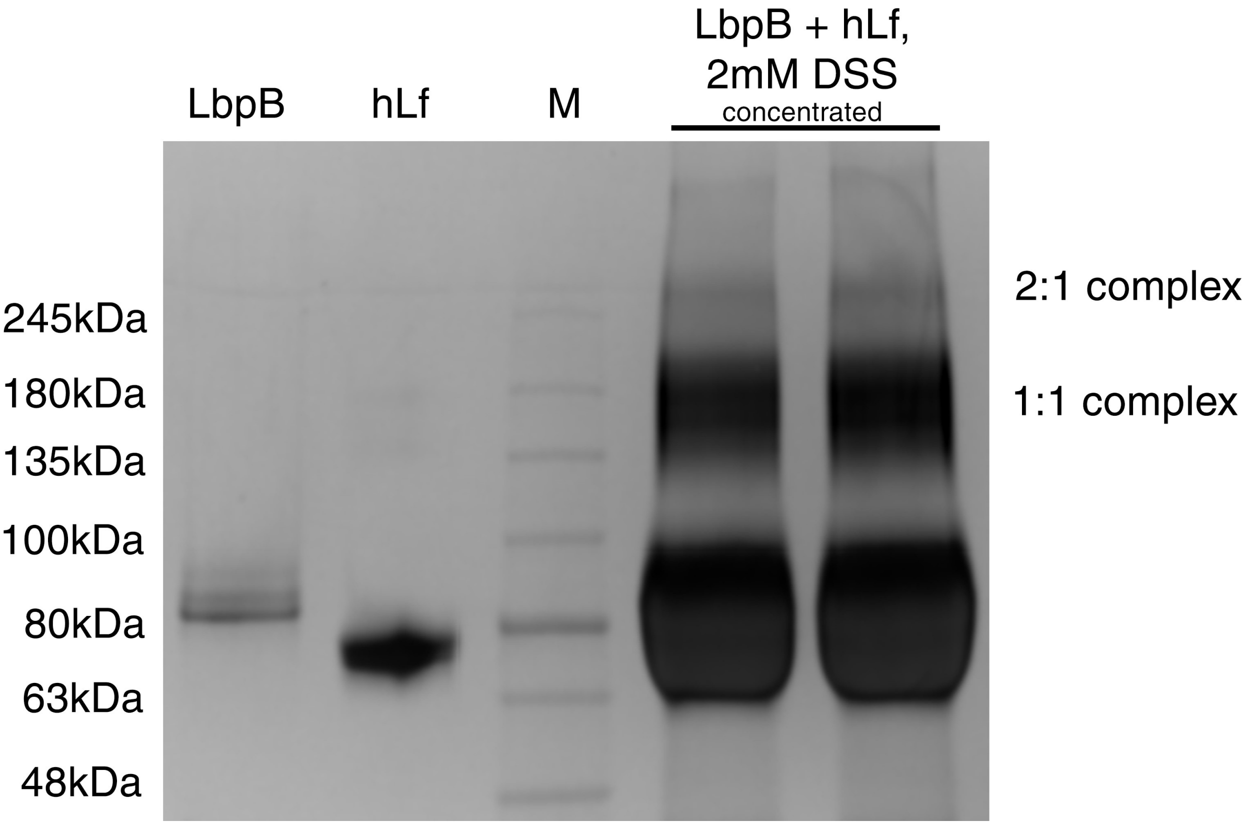

S4 Fig. SDS-PAGE of *LbpB:hLf complex populations.* TEV-cleaved LbpB and hLf were purified and loaded in lanes 1 and 2, respectively. An incubation of these two proteins in equal molar concentrations was then crosslinked with 2mM DSS. Crosslinked protein was concentrated and loaded in duplicate beside the marker (lane 3) in lanes 4 and 5. Appearance of a ~160kDa 1:1 complex, and ~245 kDa 2:1 complex are indicated.
