# Supplementary material for: Lactoferrin binding protein B – a bi-functional bacterial receptor protein
Source: PLoS Pathog. 2017 Mar 3;13(3):e1006244. doi: 10.1371/journal.ppat.1006244 (PMC5352143; doi:10.1371/journal.ppat.1006244)
Supplement: S5 Fig — Difference in deuteration between (a) hLf-LbpB complex and free hLf and (b) hLf-LbpB complex and LbpB, plotted as a function of protein sequence. A reduction in deuteration, resulting from stabilization upon binding is shown in blue. Destabilization is shown in red. Peptides for which no significant change in deuteration was observed are shown in grey (p < 0.05). Dashed grey lines indicate the 2x SD deviation cut-off based on the error in all non-significant measured deuteration values. (PDF) [file ppat.1006244.s005.pdf]

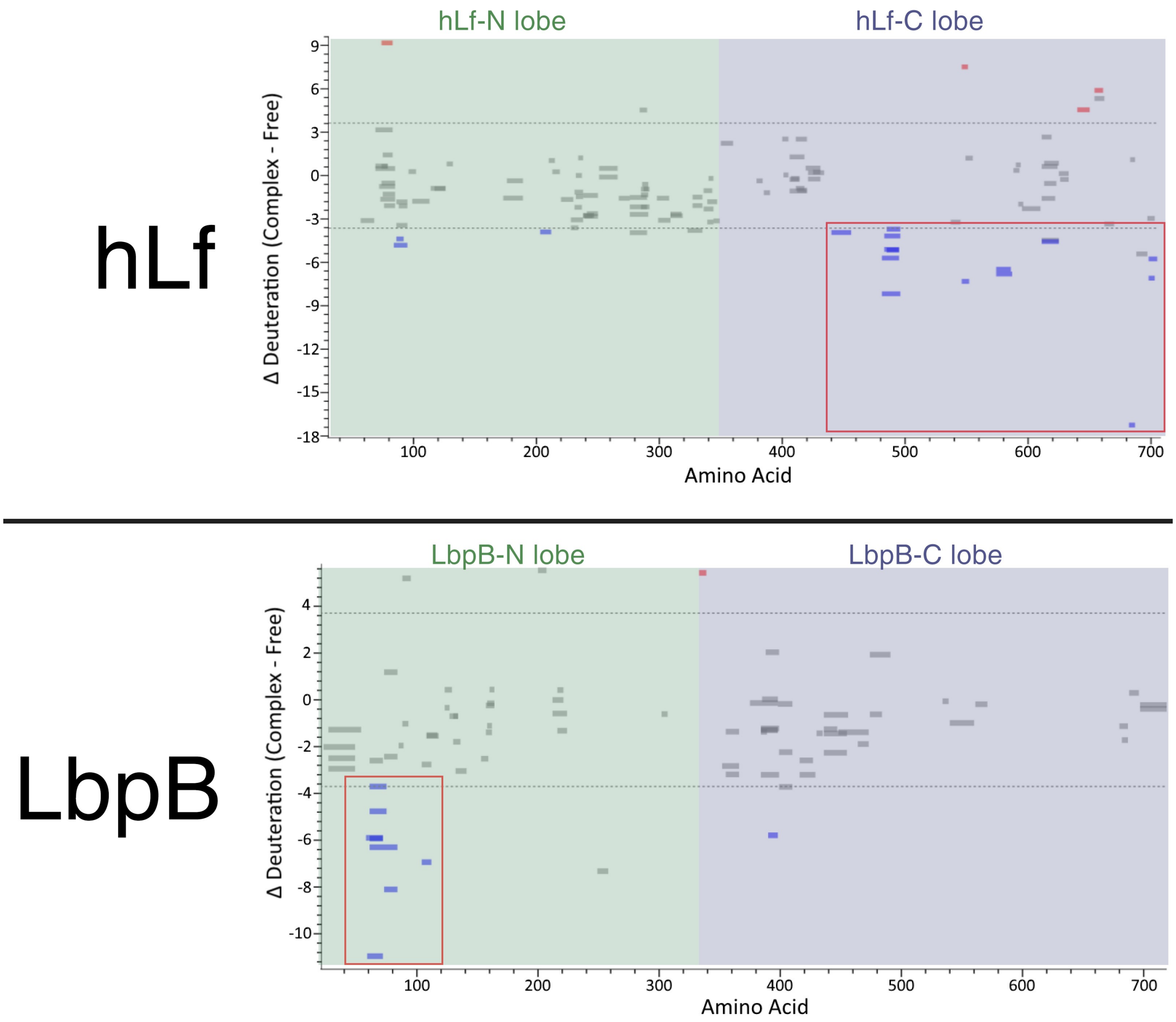

S5 Fig. HX-MS Woods plots for the interaction between hLf and LbpB. Difference in deuteration between (a) hLf-LbpB complex and free hLf and (b) hLf-LbpB complex and LbpB, plotted as a function of protein sequence. A reduction in deuteration, resulting from stabilization upon binding is shown in blue. Destabilization is shown in red. Peptides for which no significant change in deuteration was observed are shown in grey ($p < 0.05$). Dashed grey lines indicate the 2x SD deviation cut-off based on the error in all non-significant measured deuteration values.
