# Supplementary material for: Lactoferrin binding protein B – a bi-functional bacterial receptor protein
Source: PLoS Pathog. 2017 Mar 3;13(3):e1006244. doi: 10.1371/journal.ppat.1006244 (PMC5352143; doi:10.1371/journal.ppat.1006244)
Supplement: S6 Fig — (A) Docked model of LbpB-N (PDB entry 4U9C, filled in with Swiss-Model) against diferric hLf (PDB entry 2BJJ) using XL-MS constraints. Binding interface is noted with a translucent gray rectangle. (B) Crystal structure of the TbpB-N:hTf-C interaction from Neisseria meningitidis M982 (PDB entry 3VE1). (C) Alignment of docked model from (A) with crystal structure from (B). (D) Solid phase binding assay of WT and mutant LbpBs binding hLf at pH 5.9 and 7.4. (PDF) [file ppat.1006244.s006.pdf]

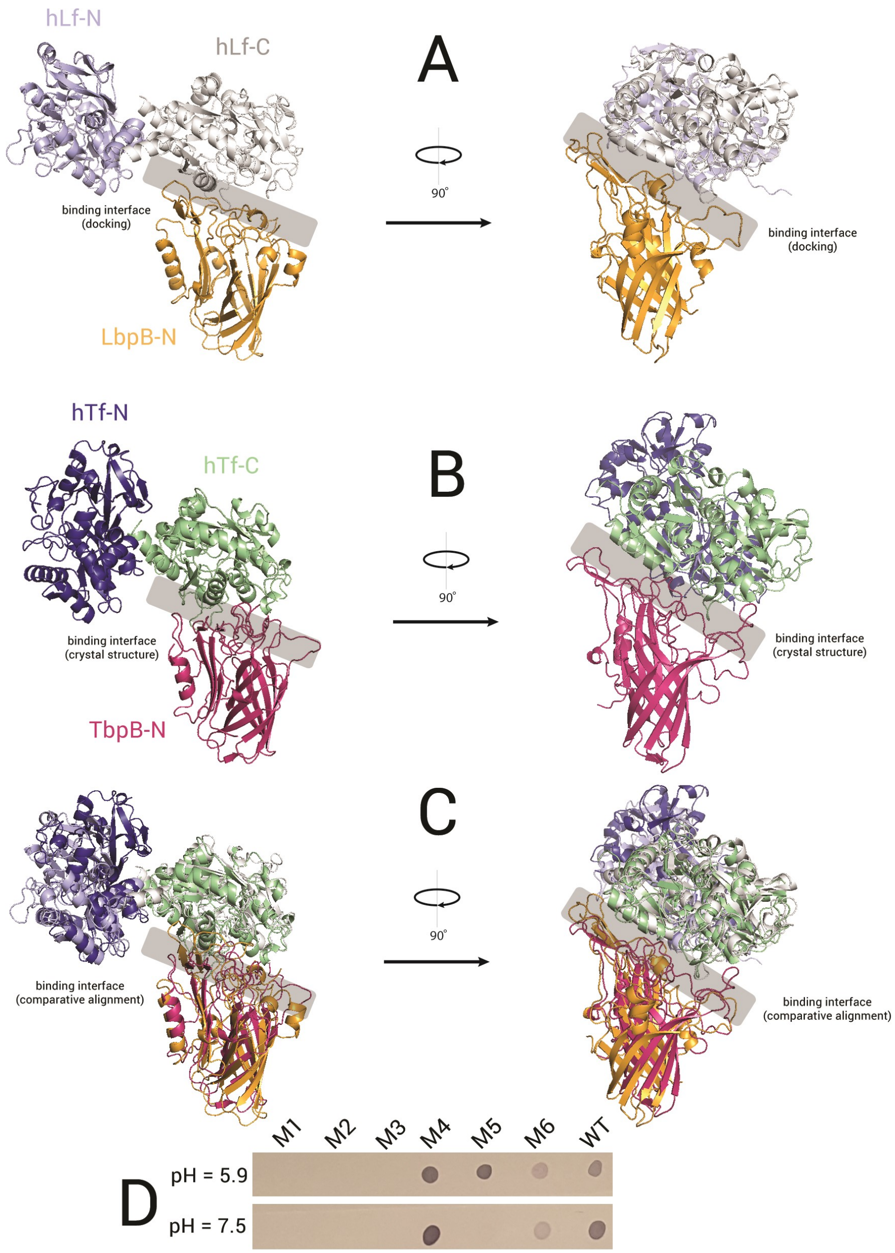

S6 Fig. *Predicted complex structure of LbpB:hLf and mutagenesis studies.* (A) Docked model of LbpB-N (PDB entry 4U9C, filled in with Swiss-Model) against diferric hLf (PDB entry 2BJJ) using XL-MS constraints. Binding interface is noted with a translucent gray rectangle. (B) Crystal structure of the TbpB-N:hTf-C interaction from *Neisseria meningitidis* M982 (PDB entry 3VE1). (C) Alignment of docked model from (A) with crystal structure from (B). (D) Solid phase binding assay of WT and mutant LbpBs binding hLf at pH 5.9 and 7.4.
