# Supplementary material for: Lactoferrin binding protein B – a bi-functional bacterial receptor protein
Source: PLoS Pathog. 2017 Mar 3;13(3):e1006244. doi: 10.1371/journal.ppat.1006244 (PMC5352143; doi:10.1371/journal.ppat.1006244)
Supplement: S1 Table — * Inter-lobe crosslink. **Low–medium confidence crosslink. (PDF) [file ppat.1006244.s007.pdf]

S1 Table. **Intra-protein crosslinks for N.m. LbpB (MC58).**

Lysine A	Lysine B	Distance (Å)	Color	Region
K199	K299	15.0	Orange	N cap – N cap
K63	K82	19.8	Blue	N cap – N handle
K103	K84	20.9	Blue	N handle – N handle
K101	K447	31.1	Blue – Red	N handle – C loops *
K370	K447	27.3	Red	C handle – C loops
K379	K563	9.5	Magenta	C loops – C loops
K701	K563	5.4	Magenta	C loops – C loops
K342	K353	27.1	Light Blue	N barrel – Lobe linker
K344	K265	22.7	Cyan	N barrel – N barrel
K118	K265	21.0	Cyan	N handle – N barrel
K231	K258	19.0	Cyan	N barrel – N cap
K435	K410	11.5	Yellow	C handle – C handle
K397	K410	11.5	Yellow	C handle – C handle
K573	K410	15.7	Yellow	C barrel – C handle
K26	K410	22.3	White	Early anchor peptide – C barrel
K26	K287	50.7	White	Early anchor peptide – N barrel**

** Inter-lobe crosslink*

***Low – medium confidence crosslink*
