# Supplementary material for: Lactoferrin binding protein B – a bi-functional bacterial receptor protein
Source: PLoS Pathog. 2017 Mar 3;13(3):e1006244. doi: 10.1371/journal.ppat.1006244 (PMC5352143; doi:10.1371/journal.ppat.1006244)
Supplement: S2 Table — * Inter-lobe crosslink. (PDF) [file ppat.1006244.s008.pdf]

S2 Table. **Intra-protein crosslinks for N.m. TbpB (B16B6).**

Lysine A	Lysine B	Distance (Å)	Color	Region
K355	K565	11.4	Orange	C handle – C barrel
K442	K480	12.6	Blue	C barrel – C barrel
K154	K105	16.0	White	N handle – N handle
K95	K131	9.0	Light Blue	N cap – N cap
K62	K194	16.1	Red	N cap – N cap
K77	K367	16.3	Yellow	N handle – C handle *
K77	K310	11.9	Yellow	N handle – N barrel
K164	K235	10.9	Magenta	N barrel – N barrel
K273	K235	10.3	Magenta	N barrel – N barrel
K279	K235	18.6	Magenta	N cap – N barrel
K252	K235	25.7	Magenta	N cap – N barrel

** Inter-lobe crosslink*
