# Supplementary material for: Lactoferrin binding protein B – a bi-functional bacterial receptor protein
Source: PLoS Pathog. 2017 Mar 3;13(3):e1006244. doi: 10.1371/journal.ppat.1006244 (PMC5352143; doi:10.1371/journal.ppat.1006244)
Supplement: S3 Table — (PDF) [file ppat.1006244.s009.pdf]

S3 Table. Intra-protein crosslinks for N.m. MBP-LbpB-C-lgsm (MC58).

Lysine A	Lysine B	Distance (Å)	Color	Region
K447	K563	21.3	Magenta	β-handle tip – β-barrel tip
K421	K563	29.3	Magenta - Yellow	β-handle tip – β-barrel tip
K421	K573	25.1	Yellow	β-barrel tip - β-handle tip
K421	K435	20.8	Yellow - Red	β-handle - β-handle
K410	K397	13.1	Red	β-handle - β-handle
K410	K435	10.9	Red	β-handle - β-handle
K410	K364	27.8	Red	β-handle - β-handle
K364	K536	12.7	Red	β-handle - β-handle
K547	K712	15.0	Blue	β-barrel rear - β-barrel rear
